# Biological evaluation of both enantiomers of fluoro-thalidomide using human myeloma cell line H929 and others

**DOI:** 10.1371/journal.pone.0182152

**Published:** 2017-08-01

**Authors:** Etsuko Tokunaga, Hidehiko Akiyama, Vadim A. Soloshonok, Yuki Inoue, Hideaki Hara, Norio Shibata

**Affiliations:** 1 Department of Nanopharmaceutical Sciences, Nagoya Institute of Technology, Nagoya, Japan; 2 Faculty of Medical Technology, Fujita Health University, Toyoake, Japan; 3 Department of Organic Chemistry I, Faculty of Chemistry, University of the Basque Country UPV/EHU, San Sebastián, Spain; 4 IKERBASQUE, Basque Foundation for Science Maria Diaz de Haro, Bilbao, Spain; 5 Molecular Pharmacology, Department of Biofunctional Evaluation, Gifu Pharmaceutical University, Gifu, Japan; Duke University School of Medicine, UNITED STATES

## Abstract

Over the last few years, thalidomide has become one of the most important anti-tumour drugs for the treatment of relapsed-refractory multiple myeloma. However, besides its undesirable teratogenic side effect, its configurational instability critically limits any further therapeutic improvements of this drug. In 1999, we developed fluoro-thalidomide which is a bioisostere of thalidomide, but, in sharp contrast to the latter, it is configurationally stable and readily available in both enantiomeric forms. The biological activity of fluoro-thalidomide however, still remains virtually unstudied, with the exception that fluoro-thalidomide is not teratogenic. Herein, we report the first biological evaluation of fluoro-thalidomide in racemic and in both (*R*)- and (*S*)-enantiomerically pure forms against (*in vitro*) H929 cells of multiple myeloma (MM) using an annexin V assay. We demonstrate that all fluoro-thalidomides inhibited the growth of H929 MM cells without any *in-vivo* activation. Furthermore, we report that the enantiomeric forms of fluoro-thalidomide display different anti-tumour activities, with the (*S*)-enantiomer being noticeably more potent. The angiogenesis of fluoro-thalidomides is also investigated and compared to thalidomide. The data obtained in this study paves the way towards novel pharmaceutical research on fluoro-thalidomides.

## Introduction

**Fig 1:**
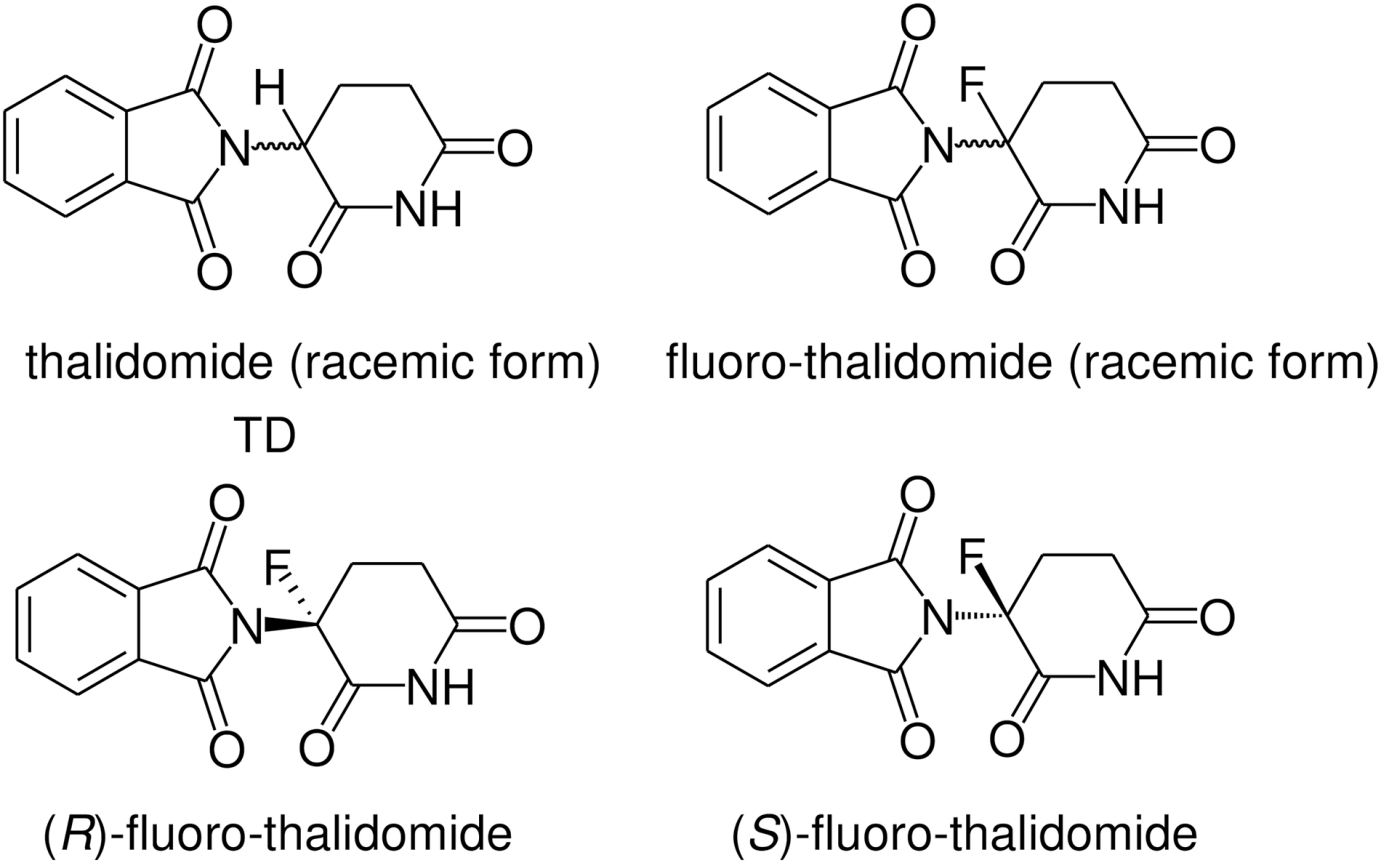
Structures of thalidomide (racemic form), fluoro-thalidomide (racemic form), and its (*R*)- and (*S*)- enantiomers, (*R*)-fluoro-thalidomide and (*S*)-fluoro-thalidomide.

Thalidomide (Fig 1) is a notorious drug, due to the significant socio-scientific impact it had on nearly every sector of the healthcare industry. When it first introduced to the market in Germany on October 1, 1957, it was heralded as a "wonder drug" for a multitude of minor health disorders such as nausea, fatigue, insomnia, coughs, colds and headaches. Four years later, it was banned, leaving a trail of demise and misery for tens of thousands of lives [1, 2]. The thalidomide tragedy precipitated a major paradigm shift in pharmacology, leading to the modern concepts of pharmacokinetics and the development of governmental structures responsible for rigorous drug approval and monitoring systems around the world [3]. One of the reasons that thalidomide was considered as a “totally safe drug” was its extraordinary low toxicity [4]. This unique property for a synthetic drug, combined with a diverse range of biological activity and relatively low cost of production, continued to attract the interest of thalidomide researchers as a promising therapeutic candidate [5]. Thus, despite its notorious history, in 1998 and 1999, respectively, the US Food and Drug Administration approved thalidomide for use in the treatment of erythema nodosum leprosum (ENL) and multiple myeloma (MM) [6]. Furthermore, the clinical success of thalidomide led to the development and marketing of several of its analogues, most notably, lenalidomide [7] and pomalidomide [8]. Considering the well-established role of fluorine in the development of modern drugs [9–13], the synthesis and biological evaluation of fluorine-containing analogues of thalidomide is likely to have great pharmaceutical potential. Among the known fluorinated derivatives of thalidomide [14–20], fluoro-thalidomide (Fig 1) [19–22] is of particular interest for at least two apparent reasons. First, it is well-known that the fluorine-for-hydrogen substitution is bioisosteric, enzymatically indistinguishable, and commonly used in the medicinal/bioorganic chemistry research [23–25]. Consequently, from the standpoint of geometric and stereochemical requirements, thalidomide and fluoro-thalidomide are ultimately close analogues. Of course, it should be kept in mind that bond polarization, polarity and charge distribution are different in these compounds [25]. Second, in sharp contrast to thalidomide [26], fluoro-thalidomide has a quaternary stereogenic centre that allows the preparation and study of (*R*)-fluoro-thalidomide and (*S*)-fluoro-thalidomide enantiomerically pure forms. This issue of configurational instability is at the very core of thalidomide’s problem. It is commonly accepted that only one of thalidomide’s enantiomers, (*S*)-thalidomide, possesses teratogenic activity while the other, (*R*)-thalidomide, has the desired therapeutic effects [27]. However, the assumption that thalidomide’s enantiomers possess different bioactivities, while very probable, cannot be unequivocally established due to its rapid *in vivo* racemization rate [28]. Consequently, there has been a longstanding significant interest in the syntheses and biological studies of configurationally stable thalidomide analogues [29–31]. In this regard, it is interesting to note that a recent study established [32] that thalidomide and its fluoro-derivative, fluoro-thalidomide possess a high magnitude of self-disproportionation of enantiomers (SDE) [33–35] when assessed by achiral chromatography. Taking into account that the SDE phenomenon is related to the ability of a chiral compound to form homo/hetero-chiral associations [36–38], one can expect that the enantiomers of thalidomide and fluoro-thalidomide might show an explicit preference for the development of homo- or hetero-chiral interactions with chiral biological receptors and, therefore, different bioproperties or bioactivities. While fluoro-thalidomide was first synthesized in 1999 by our group [21], its biological activity is still virtually unstudied. Thus, there are only preliminary reports on its tumour necrosis factor α (TNF-α) suppressive properties [19–21] and teratogenicity [39]. It is interesting to note that (*S*)-fluoro-thalidomide was found to be more potent than (*R*)-fluoro-thalidomide in inhibiting lipopolysaccharide (LPS)-induced TNF-α production in human blood leucocytes [21]. On the other hand, no significant differences between (*S*)-fluoro-thalidomide and (*R*)-fluoro-thalidomide were observed in *in vivo* biological activity of 5,6-dimethylxanthenone-4-acetic acid-induced TNF-α activity in serum and tumour tissue. Very importantly, in 2011 [39], fluoro-thalidomide was found to be non-teratogenic as the racemic form, i.e., a mixture of (*S*)-fluoro-thalidomide and (*R*)-fluoro-thalidomide, reemphasising the importance of studying the biological activity of fluoro-thalidomide and its practical potential. Given that the treatment of multiple myeloma (MM) is one of the most important therapeutic applications of thalidomide the corresponding study of fluoro-thalidomide seems to be of paramount significance. Taking advantage of the recently developed convenient and scalable procedure for the preparation of enantiomerically pure (*R*)-fluoro-thalidomide and (*S*)-fluoro-thalidomide [22], we decided to initiate a systematic study of the biological activity of racemic fluoro-thalidomide and its enantiomers (*R*)-fluoro-thalidomide and (*S*)-fluoro-thalidomide for multiple myeloma. The angiogenesis of fluoro-thalidomides is also investigated and compared to thalidomide.

## Material and methods

### Materials

Cytosine β-D-arabinofuranoside was purchased from Sigma-Aldrich, St. Louis, USA. It was used at a concentration of 10 or 20 μg/mL, as a positive control for inducing apoptosis. All other chemicals were of biochemical grade unless otherwise indicated. Thalidomide was prepared according to a reported method but using racemic ornithine [40]. Fluoro-thalidomide, (*R*)-fluoro-thalidomide and (*S*)-fluoro-thalidomide were prepared according to a previously published method [22]. They are optically pure, as was confirmed by commonly used spectroscopic and chromatographic techniques. The copies of ^19^F NMR, ^1^H NMR, and HPLC analyses of the samples may be found in the supporting information.

### Cell culture (for antitumor activity assay)

H929, a human IgA-producing cell line (DS Pharma Biomedical EC95050415; Osaka, Japan), Oda, a human IgD-producing cell line (Immuno-Biological Laboratories 37054; Maebashi, Japan), U937, a human histiocytic lymphoma cell line (DS Pharma Biomedical EC85011440; Osaka, Japan), and AGLCL, a human normal B cell line (DS Pharma Biomedical EC89120566; Osaka, Japan), were grown in RPMI 1640 medium (Sigma-Aldrich, Steinheim, Germany) supplemented with 10% inactivated fetal bovine serum (FBS; Equitech-Bio Inc., Kerrville, TX USA), 100 U/mL of penicillin, and 100 μg/mL of streptomycin (GIBCO, Carlsbad, CA USA) at 37°C with 5% CO_2_ in a humidified atmosphere.

### Assessment of apoptosis by annexin V and propidium iodide staining

To quantify apoptotic cells, an Early Apoptosis Detection kit (MBL, Nagoya, Japan) was used. The substrates thalidomide, fluoro-thalidomide, (*R*)-fluoro-thalidomide, (*S*)-fluoro-thalidomide or cytosine β-D-arabinofuranoside were added separately to RPMI1640 medium containing each cell type at a concentration of 10 or 20 μg/mL for 24 or 48 h. Cells were centrifuged at 2,000 rpm for 5 min at 4°C then suspended in 500 μL of binding buffer. Each sample containing treated cells was mixed with 5 μL of fluorescein isothiocyanate (FITC)-conjugated annexin V solution and 5 μL of propidium iodide (PI) solution at room temperature (RT) for 5 min in the dark. Cells were then analyzed by a fluorescence-activated cell sorter (FACSCalibur; Becton Dickinson, Sparks, MD USA).

### MTT assay

The 3-(4,5-dimethylthiazol-2-yl)-2,5-diphenyltetrazolium bromide (MTT) assay was investigated by evaluating cell viability using an MTT cell proliferation assay kit (Cayman Chemical Co., Ann Arbor, MI USA). H929 cells were incubated with or without thalidomide, fluoro-thalidomide, (*R*)-fluoro-thalidomide, (*S*)-fluoro-thalidomide or cytosine β-D-arabinofuranoside at a concentration of 20 μg/mL for 24 h. These treated cells were seeded at a density of 1×10^5^ cells/well in 100 μL of RPMI1640 medium in 96-well plates (Becton and Dickinson, Franklin Lakes, USA) and cultured at 37°C for 24 h. 10 μL of MTT reagent was added to each well. Cells were mixed gently, then incubated at 37°C in a 5% CO_2_ incubator. After 3 h incubation, the culture medium was aspirated and 100 μL of crystal-dissolving solution (dilute hydrochloric acid solution with sodium dodecyl sulfate (SDS)) was added to each well and mixed. Then, optical density was measured at 550 nm using a microplate reader (BIO-RAD, Benchmark, Hercules, CA USA).

### Morphological observations

H929 cells (1×10^5^ cells/mL) were first treated with or without thalidomide, fluoro-thalidomide, (*R*)-fluoro-thalidomide, (*S*)-fluoro-thalidomide or cytosine β-D-arabinofuranoside for 24 h at a concentration of 20 μg/mL. The cells (1×10^4^ cells) were then deposited on glass slide (20 mm^2^) using a cytospin centrifuge at 750 rpm for 5 min. The glass slides were fixed with Wright’s solution (Merck, Darmstadt, Germany) and stained with Giemsa solution (Merck) for observing the morphology under optical microscope (at ×400 magnifications) and the presence or absence of apoptotic bodies.

### Measurement of caspase-3, -8 and -9 activities

The activities of caspase-3, -8 and -9 were measured by a Caspase-Glo assay kit (Promega, Madison, WI USA) using DEVD-, LETD- and LEHD-aminoluciferin, respectively, as substrates, according to the manufacturer’s instructions. Briefly, a 100-μL aliquot of H929 cells (1×10^4^ cells/mL) was incubated in a 96-well plate with or without thalidomide, fluoro-thalidomide, (*R*)-fluoro-thalidomide, (*S*)-fluoro-thalidomide or cytosine β-D-arabinofuranoside for 24 h at a concentration of 20 μg/mL. A 100-μL aliquot of each caspase reagent was added and mixed, incubated for 1 h and measured by a luminescence assay (ARVOX, Perkin Elmer, Waltham, USA). Non-treated H929 cells was used as the negative control.

### Inhibition of apoptosis by caspase inhibitors

For the caspase inhibition assay, the following caspase inhibitors (MBL, Nagoya, Japan) were used: caspase-3/CPP32 inhibitor (Z-DEVD-FMK), caspase-8/FLICE inhibitor (Z-IETD-FMK), caspase-9/Mch6 inhibitor (Z-LEHD-FMK) or caspase-family inhibitor (VAD-FMK). The cells were pre-incubated at 37°C with RPMI1640 medium containing each inhibitor at 10 μM for 1 h, and then fluoro-thalidomide (20 μg/mL) was added to each PRMI1640 medium sample.

### Detection of Fas or cleaved PARP expression

H929 cells, treated with or without fluoro-thalidomide, (*R*)-fluoro-thalidomide, (*S*)-fluoro-thalidomide or cytosine β-D-arabinofuranoside at a concentration of 20 μg/mL were maintained at 37°C with 5% CO_2_ for 24 h. Then, the cells were centrifuged at 2,000 rpm for 5 min at 4°C, and washed once with PBS containing 2% FBS, and resuspended in 100 μL of PBS, and incubated at 4°C for 30 min either with appropriately diluted anti-human CD95 (Fas) antibody (BD Biosciences-Pharmingen, Franklin Lakes, USA) with phycoerythrin (PE), anti-cleaved PARP (poly [ADP-ribose] polymerase; Asp214) (BD Biosciences-Pharmingen) with PE or mouse isotype IgG1 conjugated (BD Biosciences-Pharmingen) with PE as a negative control. After washing the cells twice with PBS, each treated sample was analyzed using FACS Calibur (Becton Dickinson).

### Cell cycle analysis

H929 cells (1×10^6^ cells) treated with or without fluoro-thalidomide, (*R*)-fluoro-thalidomide, (*S*)-fluoro-thalidomide or cytosine β-D-arabinofuranoside for 24 h at a concentration of 20 μg/mL were collected by centrifugation at 2,000 rpm for 5 min and washed once with PBS. The cell pellet was suspended in 70% ethanol for at least 4 h. Thereafter, the fixed cells were centrifuged at 2,000 rpm for 5 min, washed once with PBS, and then centrifuged at 2,000 rpm for 5 min. The cell pellet was suspended in 40 μL of phosphate-citrate buffer [0.2 M Na_2_HPO_4_, 0.1 M C_3_H_4_(OH)(COOH)_3_: Sigma-Aldrich] and kept for 30 min at RT. After centrifuging at 2,000 rpm for 5 min, the cell pellet was suspended in 100 μL of PBS, and 1 μL of RNase A solution (10 mg/mL; Roche Diagnostics, Indianapolis, USA) was added and left for 30 min at 37°C. After centrifuging at 2,000 rpm for 5 min, the cell pellet was suspended in 1 mL of PBS, and 50 μL of PI solution (1.0 mg/mL; Sigma-Aldrich) was added and left for 30 min in the dark at RT. Finally, the stained cells were filtered through a cell strainer (BD Falcon; Bedford, MA, USA) and were analyzed using a fluorescence-activated cell sorter (FACS Calibur).

### Bcl-2 expression

The Muse^™^ Bcl-2 Activation Dual Detection kit (Merck, Japan), which includes two directly conjugated antibodies, a phospho-specific anti-phospho-Bcl-2 (Ser70)-Alexa Fluor^®^555 and an anti-Bcl-2-PECy5 conjugated antibody, was used to measure Bcl-2 phosphorylation relative to total levels of Bcl-2 expression. H929 cells, treated with or without fluoro-thalidomide, (*R*)-fluoro-thalidomide, (*S*)-fluoro-thalidomide or cytosine β-D-arabinofuranoside at a concentration of 20 μg/mL were maintained at 37°C with 5% CO_2_ for 24 h. Then, the cells were stained according to the kit’s protocol. Each treated sample was analyzed using a Muse^®^ Cell Analyzer (Merck, Japan).

### Cell culture (for angiogenesis assay)

Human umbilical vein endothelial cells (HUVECs, Kurabo, Japan) were cultured in a growth medium (HuMedia-EG2; Kurabo) at 37°C in a humidified atmosphere of 5% CO_2_ in air. The medium contained a base medium (HuMedia-EB2; Kurabo) supplemented with 2% fetal bovine serum (FBS), 10 ng/mL recombinant human epidermal growth factor (hEGF), 1 μg/mL hydrocortisone, 50 μg/mL gentamicin, 50 ng/mL amphotericin B, 5 ng/mL recombinant human basic fibroblast growth factor-B (hFGF-B) and 10 μg/mL heparin. Subconfluent monolayers of HUVECs, from passages 3 to 7, were used in the experiments.

### Tube formation assay

An angiogenesis assay kit (Kurabo) was used following the manufacturer’s protocol. HUVECs co-cultured with fibroblasts were cultivated in the presence or absence of various concentration of fluoro-thalidomide, (*S*)-fluoro-thalidomide, (*R*)-fluoro-thalidomide, or thalidomide with VEGF (10 ng/mL) at day 1, 4, 7 and 9. The fluoro-thalidomide, (*S*)-fluoro-thalidomide, (*R*)-fluoro-thalidomide, or thalidomide was dissolved with dimethyl sulfoxide (DMSO, a final concentration of DMSO was 0.1%). DMSO was added to the control groups. At day 11, cells were fixed with 70% ethanol. The cells were incubated with diluted primary antibody (mouse anti-human CD31, 1: 4000) for 1 h at 37°C, and with the secondary antibody (goat anti-mouse IgG alkaline phosphatase-conjugated antibody, 1:500) for 1 h at 37°C. Visualization was achieved using 5-bromo-4-chhloro-3indolyl phosphate/nitro blue tetrazolium. Images were obtained from five different fields (5.5 mm^2^ per field) for each well, and tube area, length, joints, and paths were measured using Angiogenesis Image Analyzer Ver.2 (Kurabo) as previously described [41, 42].

### Statistical analysis

Data were analyzed using Excel software and the Student’s *t*-test was used to assess the statistical significance between treated and untreated samples. Results are expressed as mean ±SD of three independent replicates.

## Results and discussion

Multiple myeloma (MM) is one of the common cancers of the blood, characterized by the accumulation of malignant plasma cells in the bone marrow compartment. MM is still an incurable disease with a 5-year survival rate of around 67% [43]. Taking into account that thalidomide monotherapy, or in combination with other drugs [44, 45], is one of the most common and effective treatments, we selected MM as the subject of this study.

### Assessment of apoptosis by annexin V and PI staining

**Fig 2:**
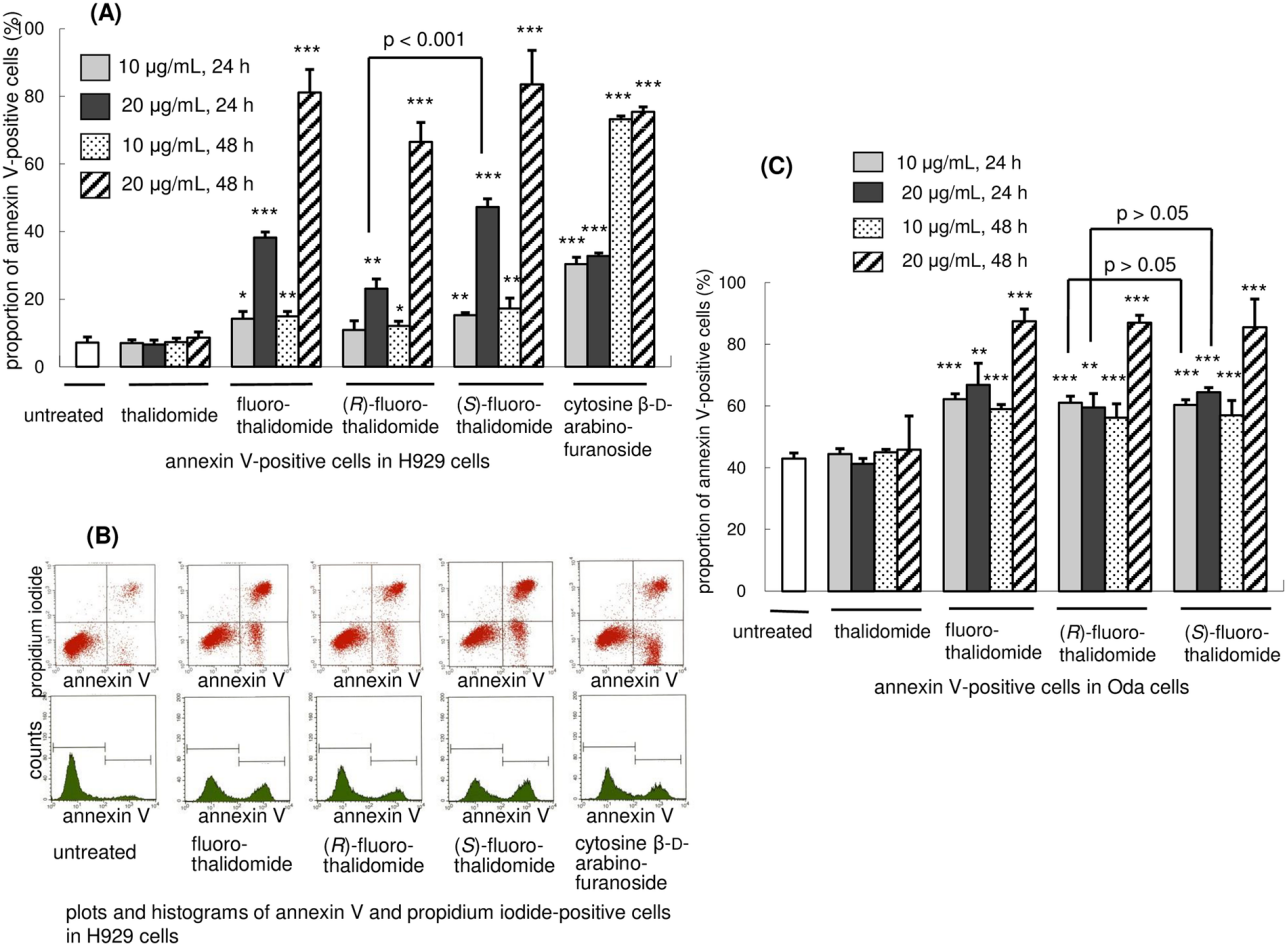
Effects of fluoro-thalidomide on H929 and Oda cells. (A) Proportion of annexin V-positive cells in H929 cells by thalidomide, fluoro-thalidomides fluoro-thalidomide, (*R*)-fluoro-thalidomide, (*S*)-fluoro-thalidomide, or cytosine β-D-arabinofuranoside (Data are shown as mean ± SD (n = 3). ***, p < 0.001 **, p < 0.01 *, p < 0.05 vs. untreated, unless otherwise noted). (B) Plots and histograms of annexin V and propidium iodide-positive cells in H929 cells after 24 h treatment with fluoro-thalidomide, (*R*)-fluoro-thalidomide, (*S*)-fluoro-thalidomide, or cytosine β-D-arabinofuranoside at a concentration of 20 μg/mL. (C) Proportion of annexin V-positive cells in Oda cells by thalidomide, fluoro-thalidomide, (*R*)-fluoro-thalidomide or (*S*)-fluoro-thalidomide.

**Fig 3:**
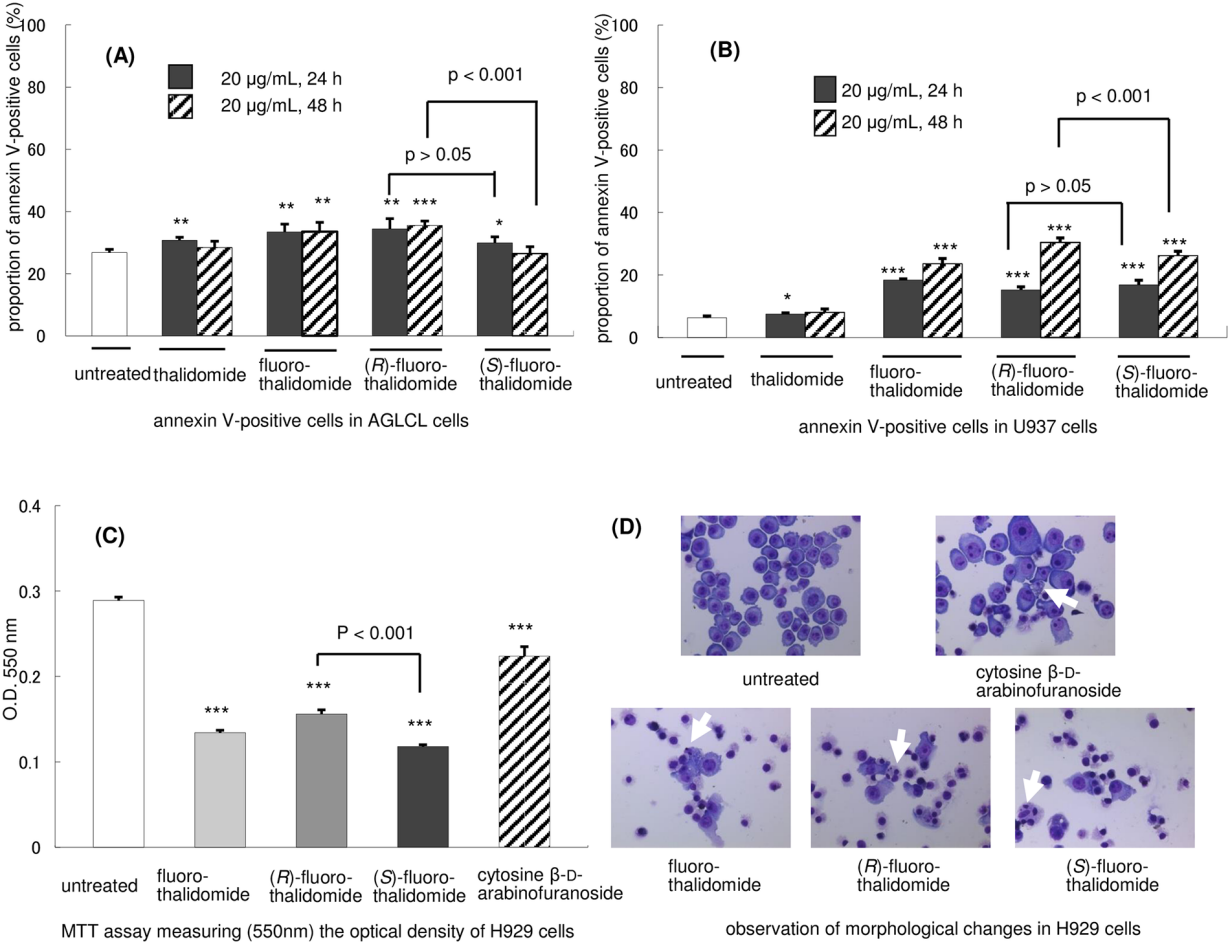
Effects of fluoro-thalidomide on H929, AGLCL and U937 cells. (A) Proportion of annexin V-positive cells in AGLCL cells, normal human B cells, by thalidomide, fluoro-thalidomide, (*R*)-fluoro-thalidomide or (*S*)-fluoro-thalidomide (Data are shown as mean ± SD (n = 3). ***, p < 0.001 **, p < 0.01 *, p < 0.05 vs. untreated, unless otherwise noted). (B) Proportion of annexin V-positive cells in U937 cells by thalidomide, fluoro-thalidomide, (*R*)-fluoro-thalidomide or (*S*)-fluoro-thalidomide. (C) MTT assay measuring (550 nm) the optical density of H929 cells treated with fluoro-thalidomide, (*R*)-fluoro-thalidomide, (*S*)-fluoro-thalidomide, or cytosine β-D-arabinofuranoside at a concentration of 20 μg/mL for 24 h. (D) Observation of morphological changes in H929 cells after treatment with fluoro-thalidomide, (*R*)-fluoro-thalidomide, (*S*)-fluoro-thalidomide, or cytosine β-D-arabinofuranoside at a concentration of 20 μg/mL for 24 h using Wright-Giemsa staining protocol. Apoptotic bodies (indicated by arrows) were observed after the treatment.

The annexin V/PI staining method is a commonly used approach to determine if cells are viable or apoptotic/necrotic based on differences in plasma membrane integrity and permeability [46]. To assess the anti-tumour activity of fluoro-thalidomide by apoptosis in H929 cells, we applied the standard annexin V/PI protocol [47] measuring the outcome by a FACS Calibur flow cytometry system. We first compared the effects of fluoro-thalidomide and thalidomide using racemic compounds, thalidomide and fluoro-thalidomide, *in vitro* on standard annexin apoptosis by flow cytometric analysis (Fig 2A). As expected from the literature [48–52], thalidomide did not induce apoptosis in the *in vitro* study (also see S1 Fig in Supporting Information). This result is accounted for by considering that thalidomide requires metabolic *in vivo* activation [48–56]. On the other hand, fluoro-thalidomide induced spontaneous apoptosis of H929 cells *in vitro*. Four separate sets of experiments were conducted next, using fluoro-thalidomide, enantiomerically pure (*R*)-fluoro-thalidomide and (*S*)-fluoro-thalidomide, as well as cytosine β-D-arabinofuranoside of known anticancer activity as a benchmark compound [57]. The results are also presented in Fig 2A. The H929 cells were treated with the indicated concentrations of racemic and enantiomerically pure fluoro-thalidomides, and cytosine β-D-arabinofuranoside for 24 h and 48 h under standard conditions. As the concentration of fluoro-thalidomides and cytosine β-D-arabinofuranoside increased, the proportions of early apoptotic cells (annexin V-positive and PI-negative) and dead cells (annexin V-positive and PI-positive) increased, whereas the proportion of viable cells (annexin V-negative and PI-negative) decreased. Additional data are shown in Fig 2B. There was a time-dependent increase in the annexin V-positive rate in all treated cells (See also S2 Fig in SI). The annexin V-positive rate of (*R*)-fluoro-thalidomide treatment was lower than fluoro-thalidomide or (*S*)-fluoro-thalidomide treatments. That is, treatment with fluoro-thalidomide, (*R*)-fluoro-thalidomide, (*S*)-fluoro-thalidomide or cytosine β-D-arabinofuranoside (20 μg/mL) resulted in 38.2, 23.1, 47.3 or 32.8% of annexin V-positive cells after 24 h, respectively. Upon incubating the cells for a further 24 hours, the percentage of annexin V-positive cells further increased to 81.1, 66.5, 83.5 or 75.4% respectively. Notably, although the absolute percentage of positive cells increased with a longer incubation time, the relative trend between fluoro-thalidomide, (*R*)-fluoro-thalidomide, (*S*)-fluoro-thalidomide or cytosine β-D-arabinofuranoside remained constant. Interestingly, was the observation that a difference was observed between the enantiomers (*R*)-fluoro-thalidomide and (*S*)-fluoro-thalidomide, where the (*S*)-enantiomer proved to be more potent to the system. There were several significant observations. Firstly, (*S*)-configured enantiomer (*S*)-fluoro-thalidomide was more active than (*R*)-enantiomer (*R*)-fluoro-thalidomide. Secondly, the observed differences in biological activities between fluoro-thalidomide, (*R*)-fluoro-thalidomide, (*S*)-fluoro-thalidomide and cytosine β-D-arabinofuranoside were much more pronounced at a higher (20 μg/mL) concentration than at 10 μg/mL. We next examined apoptosis using other cell lines, in particular Oda (a human IgD-producing cell line), U937 (a human histiocytic lymphoma cell line) and non-cancerous AGLCL (a normal human B cell line). We selected Oda since it produces IgD, and not IgA, in H929 cells. First, we measured the number of apoptotic cells in H929 cells by annexin V/PI staining. Treatment with thalidomide, fluoro-thalidomide, (*R*)-fluoro-thalidomide or (*S*)-fluoro-thalidomide resulted in 41.3, 66.9, 59.5 or 64.5%, respectively, of annexin V-positive cells after 24 h with 20 μg/mL. (*S*)-Fluoro-thalidomide was more active than (*R*)-fluoro-thalidomide (Fig 2C). After 48 h, the level apoptotic inductions of fluoro-thalidomide, (*R*)-fluoro-thalidomide or (*S*)-fluoro-thalidomide were almost the same, over 80%, while thalidomide showed the same level as untreated cells (Fig 2C). We next examined a non-cancerous AGLCL cell. After 48 hours with the treatment of 20 μg/mL, AGLCL cells showed cell death (untreated: 26.8%; thalidomide-treated: 28.4%; fluoro-thalidomide-treated 33.5%; (*R*)-fluoro-thalidomide-treated: 35.5%; (*S*)-fluoro-thalidomide-treated: 26.5%). Interestingly, while the racemic fluoro-thalidomide and (*R*)-fluoro-thalidomide induced apoptosis, the (*S*)-fluoro-thalidomide was rather non-toxic against AGLCL cells (Fig 3A). Moreover, for U937, all fluoro-thalidomide, (*R*)-fluoro-thalidomide and (*S*)-fluoro-thalidomide could induce weak apoptosis, less than 40%, even after 48 h (untreated: 6.4%; thalidomide-treated: 8.0%; fluoro-thalidomide-treated 23.6%; (*R*)-fluoro-thalidomide-treated: 30.4%; (*S*)-fluoro-thalidomide-treated: 26.2%, Fig 3B), although (*S*)-fluoro-thalidomide is weaker than fluoro-thalidomide and (*R*)-fluoro-thalidomide at a concentration of 20 μg/mL when treated for 48 h. These results indicate that when used at 20 μg/mL for 48 h, (*S*)-fluoro-thalidomide selectively induces apoptosis in multiple myeloma cells, in particular, H929 cells, while (*S*)-fluoro-thalidomide is inactive in other cells, even in non-cancerous AGLCL cells.

### MTT assay

To further assess toxicity of compounds fluoro-thalidomide, (*R*)-fluoro-thalidomide and (*S*)-fluoro-thalidomide under study, we conducted an MTT assay measuring, the optical density of the cell samples treated with fluoro-thalidomides fluoro-thalidomide, (*R*)-fluoro-thalidomide and (*S*)-fluoro-thalidomide and cytosine β-D-arabinofuranoside at a concentration of 20 μg/mL for 24 h. As can be seen from Fig 3C, optical density decreased by about 53.6% in fluoro-thalidomide, 46.0% for (*R*)-fluoro-thalidomide, 59.2% for (*S*)-fluoro-thalidomide and 22.5% in the case of cytosine β-D-arabinofuranoside, relative to untreated cells, which represented 100%. It is important to emphasize that all fluoro-thalidomides were more potent than cytosine β-D-arabinofuranoside, an observation that correlates well with the pharmaceutical potential of these fluoro-derivatives. Furthermore, in this study, we observed a dependency of bio-activity versus stereochemical properties. Thus, enantiomerically pure (*S*)-fluoro-thalidomide displayed higher potency than its racemic counterpart, i.e., fluoro-thalidomide, while (*R*)-fluoro-thalidomide was the least potent.

### Morphological changes

After H929 cells were incubated with fluoro-thalidomide, (*R*)-fluoro-thalidomide and (*S*)-fluoro-thalidomide and cytosine β-D-arabinofuranoside at a concentration of 20 μg/mL for 24 h, the morphological features, as presented in Fig 3D. The observed morphological changes indicated that the death of cells was induced by apoptosis, and not merely by toxicity. As can be seen from Fig 3D, apoptotic bodies (indicated by arrows), membrane shrinkage or chromosomal condensation were observed after the addition of fluoro-thalidomide, (*R*)-fluoro-thalidomide and (*S*)-fluoro-thalidomide and cytosine β-D-arabinofuranoside at a concentration of 20 μg/mL for 24 h, compared with untreated H929 cells.

### Caspase activity study

**Fig 4:**
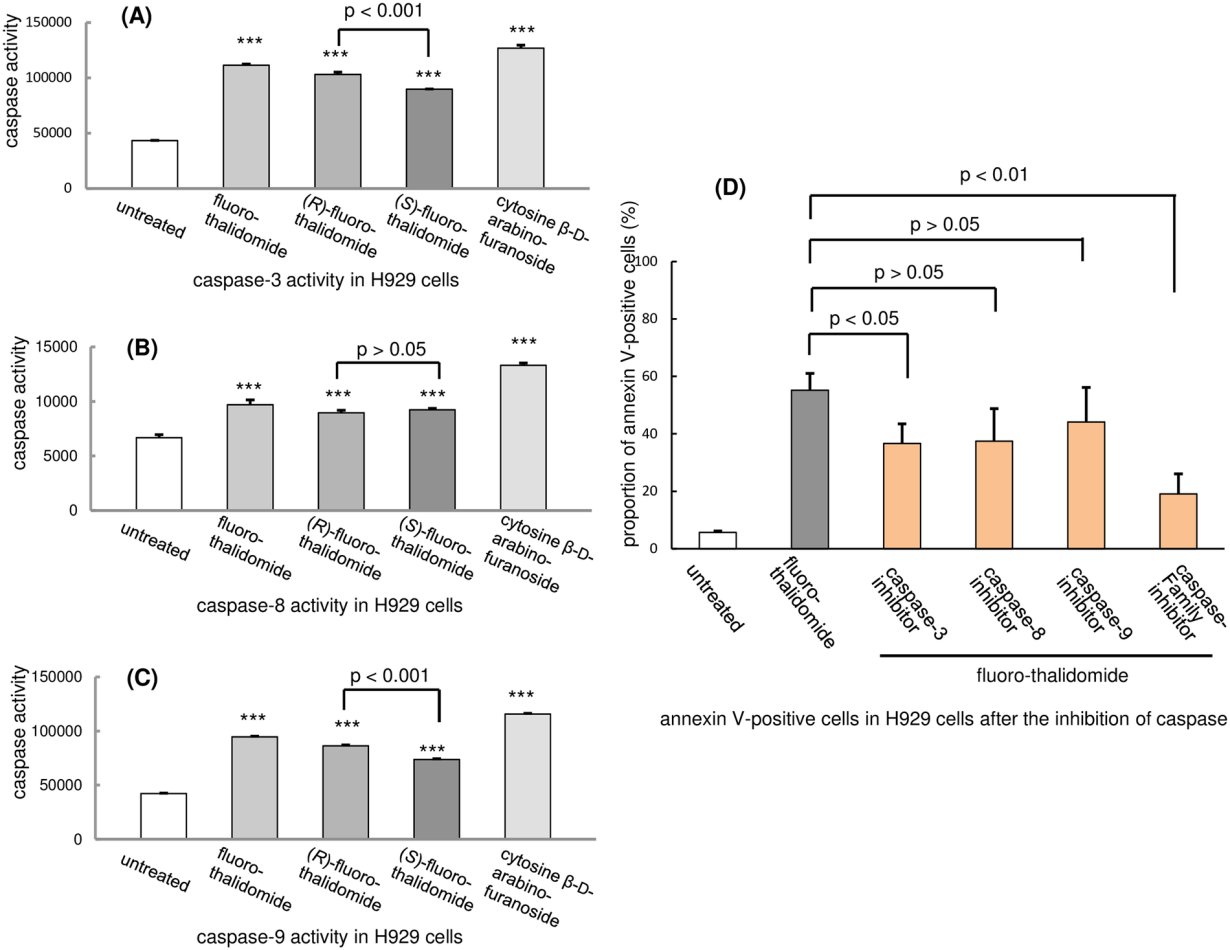
Measurement of caspase activity in H929 cells. (A) Caspase-3 activity (after treatment with fluoro-thalidomide, (*R*)-fluoro-thalidomide, (*S*)-fluoro-thalidomide or cytosine β-D-arabinofuranoside at a concentration of 20 μg/mL for 24 h. Data are shown as mean ± SD (n = 3). ***, p < 0.001 vs. untreated, unless otherwise noted). (B) Caspase-8 activity. (C) Caspase-9 activity. (D) Caspase inhibitor activity. Annexin V expression in H929 cells by fluoro-thalidomide at a concentration of 20 μg/mL for 24 h was inhibited after the addition of inhibitors of caspase-3, -8–9 and caspase-family inhibitor.

With these results in hand, we were interested in acquiring more specific data that could point to a possible mechanism of fluoro-thalidomide anti-cancerous activity. To this end, we decided to perform several experiments related to observed apoptosis pathways. It is well known that caspases are key enzymes in the initiation and regulation of cell apoptosis [58]. In particular, among the initiator caspases, caspase-9 initiates the intrinsic apoptotic pathway while caspase-8 is responsible for the extrinsic pathway [59]. Both caspase-9 and caspase-8 activate executioner caspases, such as caspase-3, -6 and -7, leading to the degradation of cellular components and, finally, apoptosis [59, 60]. With this in mind, we investigated the activities of caspase-3, -8 and -9 in H929 cells after treatment with fluoro-thalidomide, (*R*)-fluoro-thalidomide and (*S*)-fluoro-thalidomide and cytosine β-D-arabinofuranoside at a concentration of 20 μg/mL for 24 h. The results are presented in Fig 4A. The treated H929 cells showed a 2.6-, 2.4-, 2.1- and 2.9-fold increase in caspase-3, a 1.5-, 1.3-, 1.4- and 2.0-fold increase in caspase-8, and a 2.2-, 2.0-, 1.7- and 2.7-fold increase in caspase-9, respectively, relatively to the control experiment (Fig 4A). In addition, the annexin V-positive staining induced by treatment with fluoro-thalidomide was inhibited by the addition of inhibitors of caspase-3, -8 and -9, as well as a caspase-family inhibitor (Fig 4D). Considering these results, one can assume that there might be some degree of similarity between the mechanisms of biological action of fluoro-thalidomide and cytosine β-D-arabinofuranoside. Taking into account the level of activation and inhibition of caspase-3, -8 and -9 in H929 cells, we can conclude that apoptosis initiated by fluoro-thalidomide occurs via a caspase cascade.

### PARP or Fas (CD95) expression

**Fig 5:**
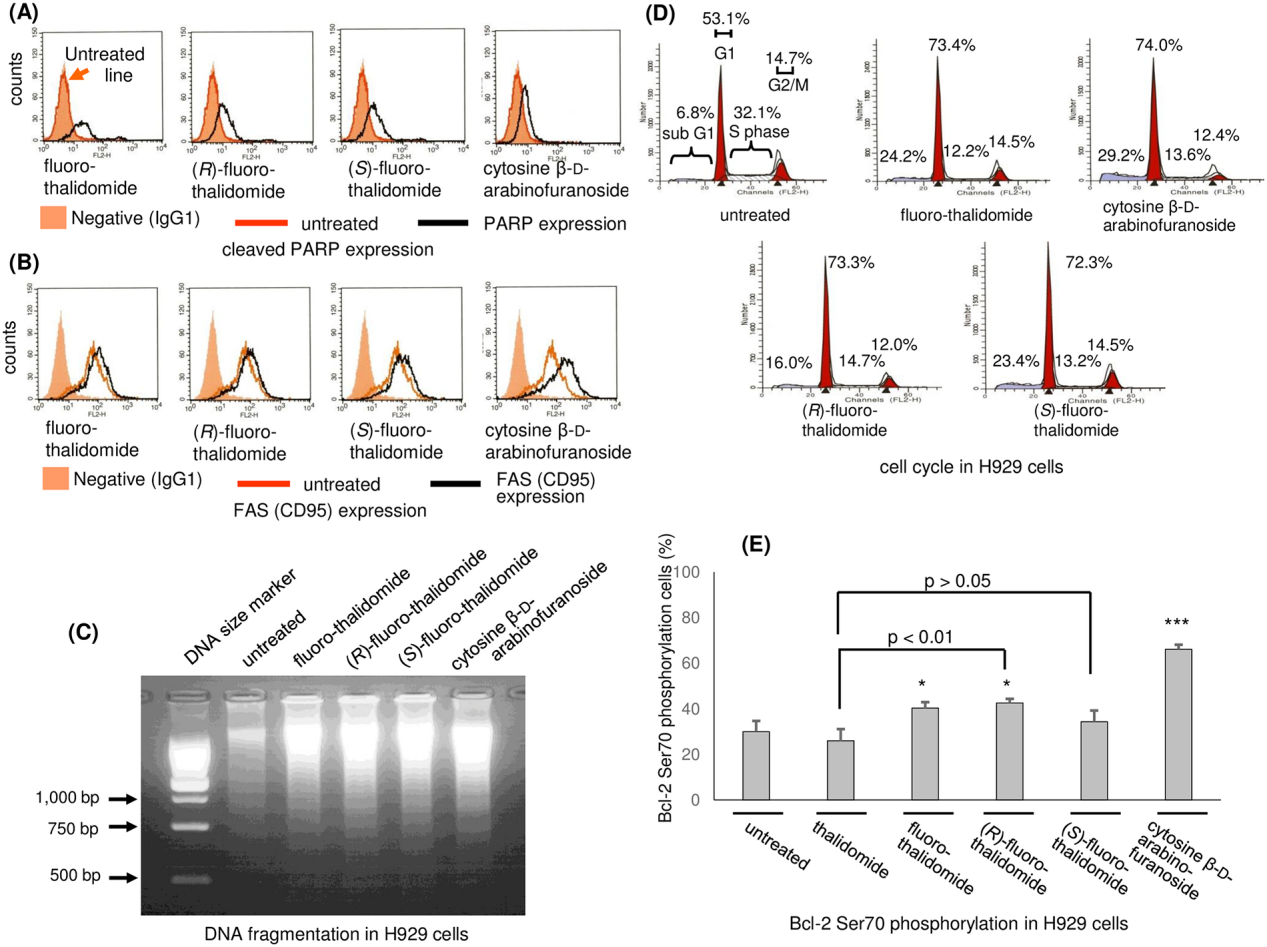
Effects of fluoro-thalidomide on H929 cells. (A) Detection of cleaved PARP expression (after treatment with fluoro-thalidomide, (*R*)-fluoro-thalidomide, (*S*)-fluoro-thalidomide or cytosine β-D-arabinofuranoside at a concentration of 20 μg/mL for 24 h unless otherwise noted). (B) Detection of Fas (CD95) expression. (C) From left to right, detection of DNA fragmentation treated with cytosine β-D-arabinofuranoside, (*S*)-fluoro-thalidomide, (*R*)-fluoro-thalidomide, fluoro-thalidomide and untreated sample by agarose gel electrophoresis compared to DNA size marker (100bp DNA Ladder, Promega, Madison, USA). (D) Cell cycle analysis. The proportion of cells in the G1, S, G2/M or sub-G1 phase. Data are shown as mean (n = 3). (E) Phosphorylation expression of Bcl-2 Ser 70. Data are shown as mean ± SD (n = 3). ***, p < 0.001 **, p < 0.01 *, p < 0.05 vs. untreated.

PARP is a large family of proteins with 17 members that are critically involved in various cellular processes, including caspase-independent apoptosis [60]. Since PARP is a target of apoptosis-associated caspases, cleavage of PARP strongly suggests caspase activation. The measurements of PARP expression in H929 cells after treatment with fluoro-thalidomides fluoro-thalidomide, (*R*)-fluoro-thalidomide and (*S*)-fluoro-thalidomide and cytosine β-D-arabinofuranoside at a concentration of 20 μg/mL for 24 h are presented in Fig 5A. Anti-cleaved PARP (Asp214) antibody detected a large fragment (89 kDa) of human PARP1 produced by caspase cleavage. In fluoro-thalidomides fluoro-thalidomide, (*R*)-fluoro-thalidomide, (*S*)-fluoro-thalidomide or cytosine β-D-arabinofuranoside-treatment experiments, cleaved PARP was detected using anti-cleaved PARP (Asp214) (Fig 5A). Furthermore, the expression of Fas antigen (CD95) [61] was also unmistakably observed (Fig 5B). However, the level of expression induced by fluoro-thalidomide, (*R*)-fluoro-thalidomide and (*S*)-fluoro-thalidomide was relatively weaker than that of cytosine β-D-arabinofuranoside. Nevertheless, the detected PARP expression and Fas (CD95) expression all strongly support that apoptosis observed in the presence of fluoro-thalidomide, (*R*)-fluoro-thalidomide and (*S*)-fluoro-thalidomide was induced by a caspase cascade [62].

### Detection of DNA fragmentation

The next set of experiments focused on the detection of DNA fragmentation, using agarose gel electrophoresis, when cells were incubated with fluoro-thalidomide, (*R*)-fluoro-thalidomide and (*S*)-fluoro-thalidomide and cytosine β-D-arabinofuranoside at a concentration of 20 μg/mL for 24 h (Fig 5C). Apoptotic DNA fragmentation was easily detectable after treatment for 24 h with fluoro-thalidomide, (*R*)-fluoro-thalidomide, (*S*)-fluoro-thalidomide or cytosine β-D-arabinofuranoside, unlike DNA of untreated samples. Once again, these data point to a similarity in the biological action of cytosine β-D-arabinofuranoside and fluoro-thalidomide, (*R*)-fluoro-thalidomide and (*S*)-fluoro-thalidomide.

### Analysis of the cell cycle

When the cell cycle was analyzed, the proportion of cells in the G1, S, G2/M or sub-G1 phase was measured in the presence of fluoro-thalidomide, (*R*)-fluoro-thalidomide, (*S*)-fluoro-thalidomide and cytosine β-D-arabinofuranoside at a concentration of 20 μg/mL for 24 h. In untreated H929 cells, 53.1±7.0% of cells were in the G1 phase, 32.1±4.5% in the S phase, 14.7±3.3% in the G2/M phase and 6.8±0.5% in the sub-G1 phase. After treatment with fluoro-thalidomide, (*R*)-fluoro-thalidomide, (*S*)-fluoro-thalidomide and cytosine β-D-arabinofuranoside, the proportion of cells in the S phase was 12.2±4.8% for fluoro-thalidomide, 14.7±3.6% for (*R*)-fluoro-thalidomide, 13.2±3.7% for (*S*)-fluoro-thalidomide or 13.6±5.7% for cytosine β-D-arabinofuranoside, and the sub-G1 phase, it was 24.2±7.9% for fluoro-thalidomide, 16.0±3.5% for (*R*)-fluoro-thalidomide 23.4±3.7% for (*S*)-fluoro-thalidomide or 29.2±8.4% for cytosine β-D-arabinofuranoside (Fig 5D). More details including in the G1 phase and the G2/M phase are also shown in Fig 5D. Both fluoro-thalidomides and cytosine β-D-arabinofuranoside induce G1 cell cycle arrest and decrease the number of cells in the S phase. Although (*R*)-fluoro-thalidomide and (*S*)-fluoro-thalidomide affect the individual phases of the cell cycle, only minor differences are observed between the two enantiomers. Interestingly, while (*R*)-fluoro-thalidomide and (*S*)-fluoro-thalidomide showed similar effects on the cell cycle, we did observe that (*S*)-fluoro-thalidomide has a more prominent effect on the sub-G1 phase in comparison to (*R*)-fluoro-thalidomide. These results are in good agreement with the results shown in Fig 2A.

### Bcl-2 expression

The internal apoptosis pathway, due to intracellular stress, involves the permeabilization of the mitochondrial outer membrane by the Bcl-2 family. The Bcl-2 family members comprise three subfamilies, Bcl-2 and its homologues, Bcl-xL and Bcl-w which strongly impede apoptosis in response to cytotoxic stimuli. Among various modes of action of Bcl-2, the phosphorylation of Ser 70 contributes to the inhibition of apoptosis. In other words, the dephosphorylation of Bcl-2 Ser 70 accelerates apoptosis [63, 64]. To examine the contribution of Bcl-2 for the apoptosis observation by fluoro-thalidomides, we investigated the expression of Bcl-2 and the phosphorylation of Ser 70 in H929 cells after treatment with fluoro-thalidomide, (*R*)-fluoro-thalidomide and (*S*)-fluoro-thalidomide. Cytosine β-D-arabinofuranoside was also examined for comparisons. H929 cells overexpressed Bcl-2, regardless of the treatment. Treatment with thalidomide, fluoro-thalidomide, (*R*)-fluoro-thalidomide, (*S*)-fluoro-thalidomide or cytosine β-D-arabinofuranoside resulted in 26.0, 40.3, 42.6, 34.3 or 66.1%, respectively of phospho-specific Bcl-2 Ser70, namely activated Bcl-2 expression, after 24 h treatment with 20 μg/mL (Fig 5E). The value was 30.0% without treatment. It should be noted that the phosphorylation of Bcl-2 Ser70 in H929 cells slightly increased after treatment with fluoro-thalidomide, (*R*)-fluoro-thalidomide or (*S*)-fluoro-thalidomide instead of dephosphorylation, while the strong apoptosis was observed (Fig 2A). However, the phosphorylation of Bcl-2 Ser70 by the treatment with (*S*)-fluoro-thalidomide was weakest compared to that of fluoro-thalidomide and (*R*)-fluoro-thalidomide which is good agreement with the fact that (*S*)-fluoro-thalidomide indicates highest apoptosis observation (Fig 2A). On the other hand, 66.1% of phosphorylation by the treatment with was observed, confirming the activation of on the Bcl-2 signaling pathway [65, 66].

### Plausible mechanism of the biological activity of fluoro-thalidomides

After considering all of the data discussed above, we propose a plausible mechanism of the biological activity of fluoro-thalidomides. We believe that two pathways are activated. In one pathway, intracellular stress, caused by fluoro-thalidomides, activates caspase-9 by adjusting mitochondrial membrane permeability via the Bcl-2 family. On the other hand, it is the death receptor pathway that is enhanced by fluoro-thalidomides, via both Fas receptor and Fas ligand expression on H929 cells. Even though the contribution of Fas ligand-Fas receptor interactions to the cytotoxic activity of these drugs remains unclear, trimerisation of the Fas receptor leads to subsequent recruitment of caspase-8 [67]. The engaged caspase-8 and caspase-9 cleave and activate further caspases, initiating a caspase cascade, ultimately leading to cell apoptosis.

### Fluoro-thalidomide, not thalidomide, promotes VEGF-induced tube formation in HUVECs

**Fig 6:**
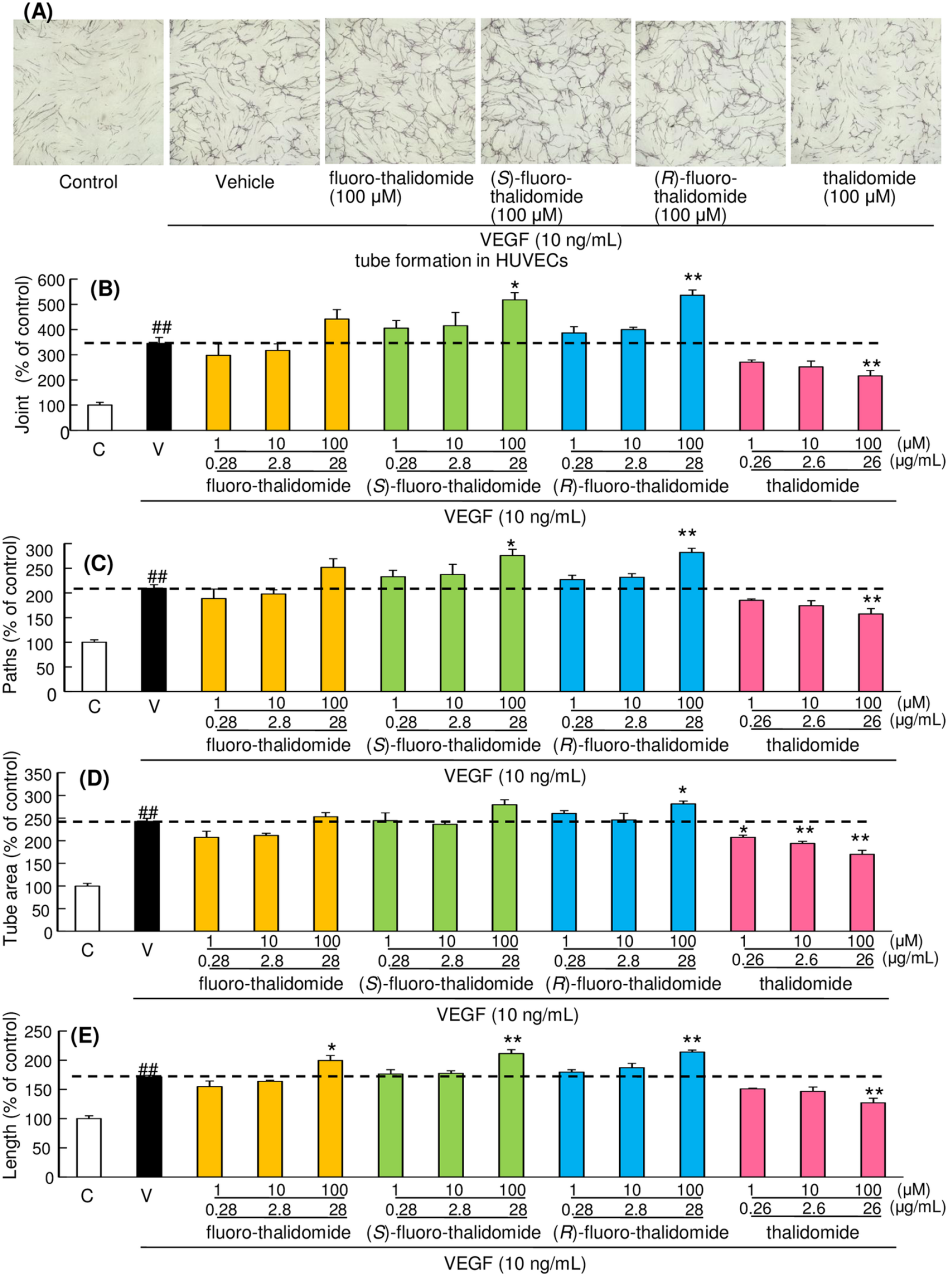
Fluoro-thalidomide not thalidomide promotes VEGF induced tube formation in HUVECs. (A) Representative photographs were shown in tube formation. Quantitative analysis of stained tube formation structures was measured by using angiogenesis imaging analyzer, version 2 in five different areas for each well. We measured joint (B), joints (B), paths (C), tube area (D), lengths (E). Data are shown as mean ± SEM (n = 3 or 4). ^##^, p < 0.01 vs. Control group, and *, p < 0.05 **, p < 0.01 vs. Vehicle-treated group.

To detect a tube formation network, we used a tube formation assay in which HUVECs and fibroblasts were co-cultured. After 11 days of incubation, HUVECs became organized into complex tubular networks exposed to 10 ng/mL of VEGF, and this effect was promoted by fluoro-thalidomide, (*S*)-fluoro-thalidomide, and (*R*)-fluoro-thalidomide addition in a concentration dependent manner. However, treatment with thalidomide decreased VEGF-induced tube formation (Fig 6A). To estimate the formation of capillary like structures, we performed a quantitative determination of the tube area, tube length, joints, and paths as indexes. We identified that exposure to 100 μM of fluoro-thalidomide, (*S*)-fluoro-thalidomide, (*R*)-fluoro-thalidomide increased HUVEC tube formation. While others have reported that thalidomide decreases HUVEC tube formation [68–70] (Fig 6B–6E), interestingly, fluoro-thalidomide did not induce similar effects. While it is widely reported that thalidomide attenuates nitric oxide-driven angiogenesis by interacting with soluble guanylyl cyclase, all fluoro-thalidomides instead induced the activation of angiogenesis. The contrast in these results can be explained by understanding the relationship between bcl-2 and angiogenesis. Previous reports have shown that activation of bcl-2 induces angiogenesis [71]. Based on these facts, and having proven that fluoro-thalidomides cause bcl-2 activation, while thalidomide in contrast de-activates bcl-2, we predict that it is this mechanism which is causing angiogenesis to be either attenuated or activated. This explanation is in good agreement with the data shown in Fig 5E. Previously, others have shown that fluoro-thalidomide is non-teratogenic [39], which due to the direct link between anti-angiogenesis and teratogenic effects is in good agreement with our results.

## Conclusion

Our initial study of the anti-tumour activity of fluoro-thalidomides against H929 revealed that a fluorine analogue of thalidomide, i.e., fluoro-thalidomide and its enantiomers are more potent *in vitro*, compared with the benchmark antitumor drug, cytosine β-D-arabinofuranoside. Moreover, the biological activity of enantiomer (*S*)-fluoro-thalidomide and fluoro-thalidomide are markedly higher than (*R*)-fluoro-thalidomide. This work presents the first study of anti-tumor activity of fluoro-thalidomides in racemic, and both (*R*) and (*S*)-enantiomerically pure forms. We demonstrate that fluoro-thalidomide, (*R*)-fluoro-thalidomide and (*S*)-fluoro-thalidomide can inhibit the growth of human multiple myeloma cell line H929 and Oda MM cells at a concentration of 20 μg/mL after treatment for 48 h. Furthermore, in sharp contrast to thalidomide, all fluoro-thalidomides do not require any metabolic activation to manifest its strong anti-tumour activity. Moreover, we report the first explicit evidence that the enantiomeric forms of fluoro-thalidomide display different anti-tumor activities in H929 MM cells. In particular, the (*S*)-enantiomer of fluoro-thalidomide, (*S*)-fluoro-thalidomide was more potent than its racemate, fluoro-thalidomide or the (*R*)-configured isomer, (*R*)-fluoro-thalidomide. (*S*)-Fluoro-thalidomide was more potent *in vitro*, when compared with cytosine β-D-arabinofuranoside. It should be noted that (*S*)-fluoro-thalidomide selectively induced apoptosis of MM cells but were inactive for other cells, including normal human B cells at a concentration of 20 μg/mL after treatment for 48 h. Moreover, all fluoro-thalidomides induce angiogenesis in contrast with the inhibition of angiogenesis by thalidomide. With the reported data of TNF-α suppressive properties and non-teratogenicity of fluoro-thalidomides, the data we disclose in this work strongly suggest that the fluoro-thalidomide would be an attractive therapeutic candidate worthy of systematic evaluation to ascertain its biological activity. Further mechanistic studies including specific ligand-binding study of fluoro-thalidomide is now under investigation.

## Supporting information

S1 Fig Effects of thalidomide on H929. Proportion of annexin V-positive cells after 48 h with 100 μg/mL or 500 μg/mL in H929. Thalidomide did not induce apoptosis in H929 cells independent of the concentration and configuration, (*R*)-thalidomide or (*S*)-thalidomide.
(TIF)

S2 Fig A time-depending change of proportion of annexin V-positive cells. Plots after 6, 12, 24 h treatment with fluoro-thalidomide at a concentration of 20 μg/mL.
(TIF)
