# Supplementary figures and images for: Biological evaluation of both enantiomers of fluoro-thalidomide using human myeloma cell line H929 and others
Source: PLoS One. 2017 Aug 1;12(8):e0182152. doi: 10.1371/journal.pone.0182152 (PMC5538663; doi:10.1371/journal.pone.0182152)

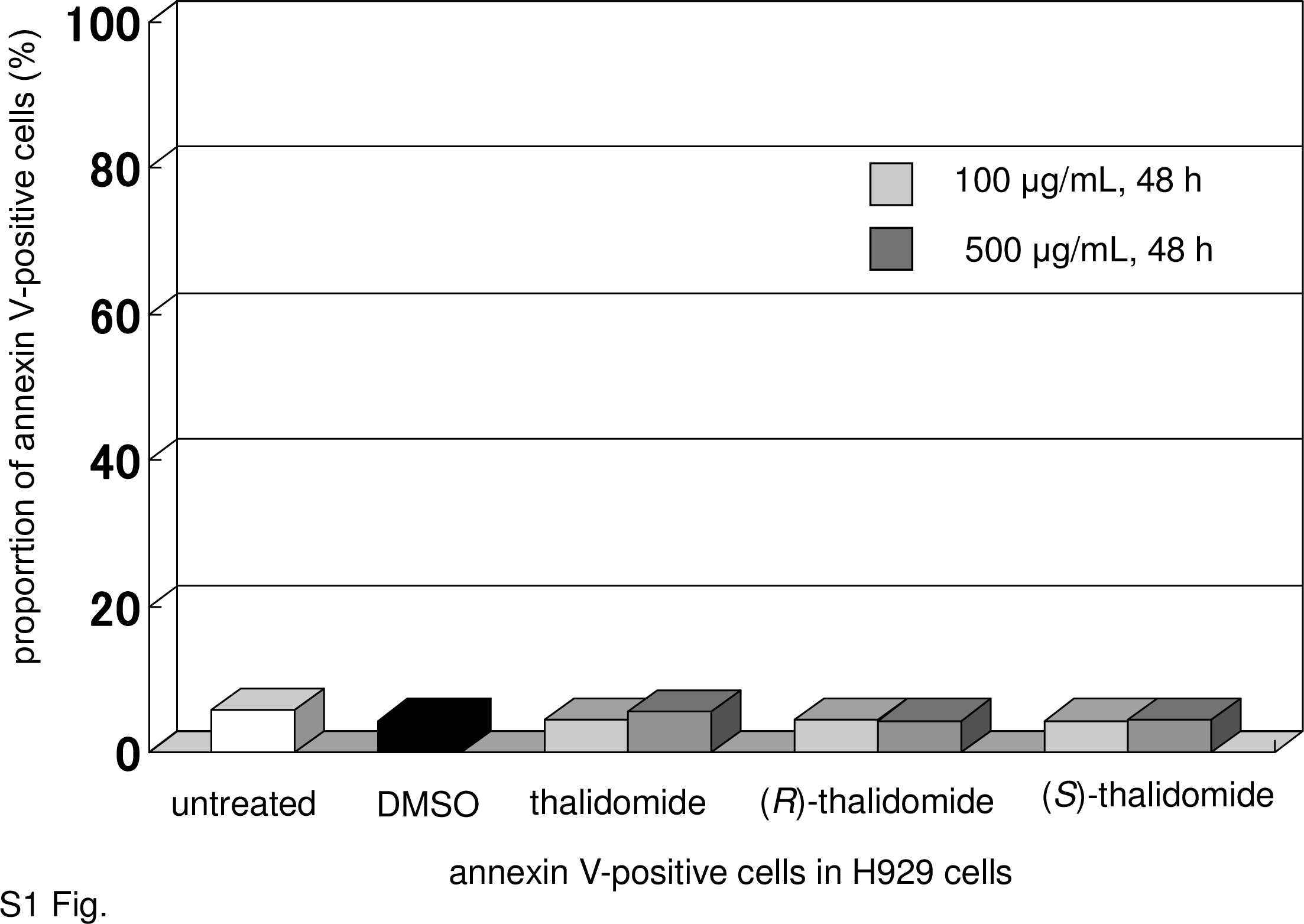

Supplement: S1 Fig — Proportion of annexin V-positive cells after 48 h with 100 μg/mL or 500 μg/mL in H929. Thalidomide did not induce apoptosis in H929 cells independent of the concentration and configuration, (R)-thalidomide or (S)-thalidomide. (TIF) [file pone.0182152.s001.tif]

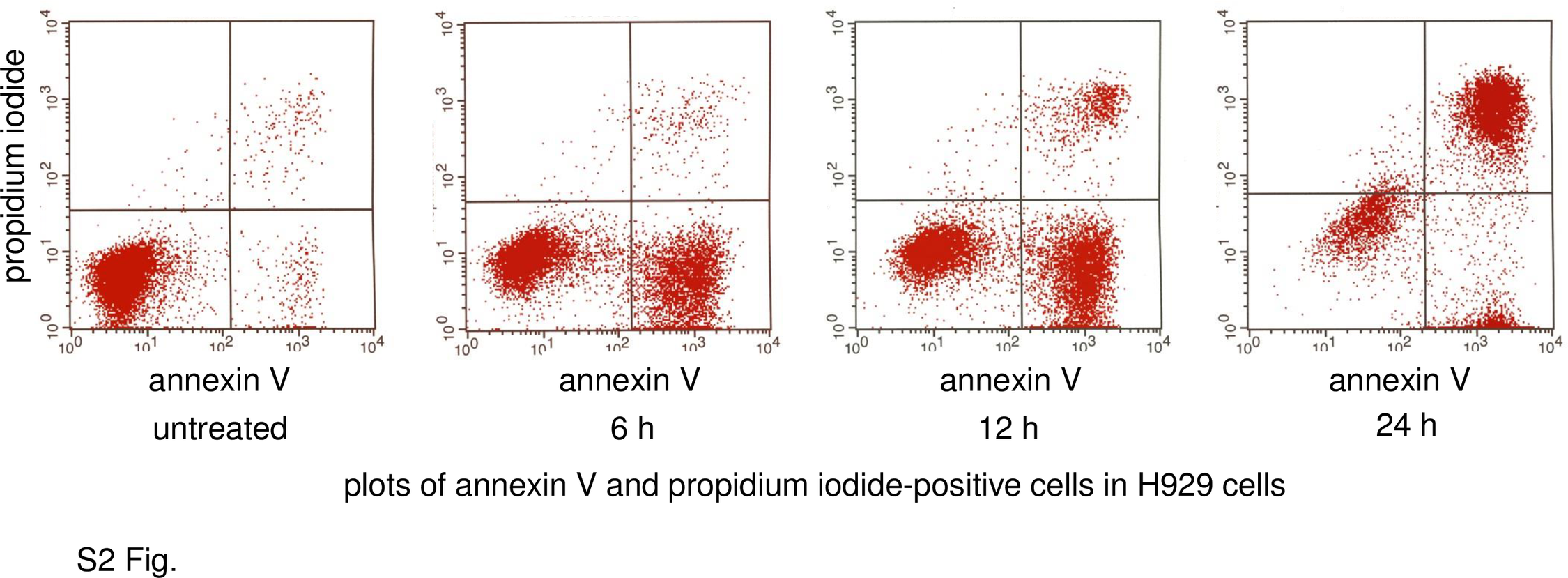

Supplement: S2 Fig — Plots after 6, 12, 24 h treatment with fluoro-thalidomide at a concentration of 20 μg/mL. (TIF) [file pone.0182152.s002.tif]
